# Enhancing Gun Violence Surveillance with Endemic-Epidemic Modeling: A Case Study in Washington, D.C.

**DOI:** 10.1007/s11524-026-01128-5

**Published:** 2026-08-17

**Authors:** Michael R. Desjardins, Samuel Roubin, Frank C. Curriero, Mallory O’Brien, Cassandra Crifasi, Daniel W. Webster

**Affiliations:** 1 Department of Epidemiology and Spatial Science for Public Health Center, Johns Hopkins Bloomberg School of Public Health, Baltimore, MD USA; 2 Department of Oncology and Sidney Kimmel Comprehensive Cancer Center, Johns Hopkins School of Medicine, Baltimore, MD USA; 3 Center for Gun Violence Solutions, Johns Hopkins Bloomberg School of Public Health, Baltimore, MD USA; 4 Center for Injury Research and Policy, Johns Hopkins Bloomberg School of Public Health, 2004 McElderry St, Baltimore, MD 21205 USA; 5 Department of Biostatistics, Johns Hopkins Bloomberg School of Public Health, Baltimore, MD USA; 6 Department of Health Policy and Management, Johns Hopkins Bloomberg School of Public Health, Baltimore, MD USA

**Keywords:** Gun violence, Spatial analysis, Social determinants of health, Endemic-epidemic modeling, Spatial statistics

## Abstract

Gun violence is a persistent public health concern in the United States, particularly in disadvantaged neighborhoods facing social and economic hardships. Washington, D.C., ranks among cities with the highest gun violence rates, incurring an estimated annual economic burden of over $2 billion. This issue is shaped not only by place-based factors but also by dynamic processes, such as local recurrence and spillover from nearby events that elevate short-term risk. This study utilized an endemic–epidemic model to disentangle contributions to background risk versus dynamic processes, analyzing approximately 24,000 firearm-related incidents in D.C. census tracts over 596 weeks from January 2014 to July 2025. Time-varying structural factors, including social vulnerability metrics and alcohol outlet access, were considered. Key findings revealed that ~ 79% of gun violence incidents were driven by endemic factors linked to long-term structural vulnerabilities, notably high alcohol outlet density and low access to essential resources. Conversely, short-term spikes in violence were influenced significantly by recent incidents within a tract, with minimal spillover effects from neighboring areas. We also mapped tracts and weeks that exceeded baseline predictions to highlight areas experiencing sustained surges in gun violence, providing valuable insights for future community alerts. These findings underscore the need to address localized issues and persistent long-term risk factors. Effective solutions must prioritize enhancing neighborhood conditions and community resources. Addressing these upstream or “root cause” factors is crucial in a public health approach to gun violence, emphasizing the importance of considering structural and social determinants, and dynamic processes influencing spikes in violence.

**Supplementary Information:**

The online version contains supplementary material available at https://doi.org/10.1007/s11524-026-01128-5.

## Introduction

Gun violence is a persistent public health crisis in the United States (U.S.) despite recent declines in firearm homicides [1]. Although firearm homicide rates in the U.S. have experienced notable fluctuations over the past four decades, the country has consistently remained an extreme outlier among high-income nations. Over the past decade, U.S. firearm homicide rates surged in 2020 (6.1 per 100,000), peaked in 2021 (6.36 per 100,000), but have declined sharply since through the end of 2025 (4.3 per 100,000) [2]. Since adolescents and young adults are disproportionately impacted, the deaths in 2023 represented an estimated 572,601 years of potential life lost [3]. For Black individuals, firearm homicides were the leading cause of death among ages 15–24 and the second leading cause of death for ages 25–34 [3, 4]. Washington, D.C. consistently ranks among 100 cities with the highest homicide rates in the nation, with an estimated annual economic burden of over $2 billion and 274 homicides reported in 2023, the largest spike in decades [5, 6].

Gun violence is most pervasive in neighborhoods characterized by racialized and concentrated social and economic disadvantage, disinvestment, and blight with more alcohol outlets and environmental toxins compared with more advantaged neighborhoods [7–10]. Neighborhood-level conditions are increasingly recognized as proximal structural drivers of violence, operating through mechanisms including routine activity patterns [11], social cohesion and collective efficacy [12], and the physical environment [10, 13, 14]. While individual-level risk factors remain important, place-based approaches offer distinct advantages for population-level surveillance and intervention, particularly given that gun violence is highly geographically concentrated in areas of sustained disadvantage [15, 16]. A national study utilizing 2015 census tract-level gun violence data found that regions with improved socioeconomic conditions experienced decreases in firearm-related homicide rates [17]; however, it did not examine non-fatal shootings or include important social determinants of health (SDoH) and place-based factors related to gun violence such as food insecurity [18], transportation access [19], alcohol outlet density [20], and even the impact of COVID-19 [21]. These omissions suggest opportunities for new research to incorporate a broader range of SDoH, enhancing our understanding of the complex factors influencing gun violence and informing effective interventions aimed at reducing violence in affected communities.

Gun violence is not only influenced by the SDoH and place-based factors, which are relatively static features of neighborhoods, but also by dynamic processes: time-dependent mechanisms whereby violent events increase the short-term risk of subsequent violence, through local recurrence (e.g., retaliatory or repeat violence) within the same area and spillover into neighboring areas such as local recurrence (e.g., retaliatory or repeat violence) and spillover from neighboring areas, whereby violent events increase the short-term risk of subsequent violence nearby [22]. This complexity presents a unique opportunity for employing spatiotemporal endemic-epidemic modeling [23], which has not yet been widely utilized in this context. While efforts have been made to apply spatiotemporal contagion models to crime and gun violence data [24, 25], these approaches have largely emphasized event clustering and prediction, with limited use of regression-based endemic–epidemic frameworks that explicitly incorporate covariates to disentangle and quantify contributions to background (endemic) risk versus dynamic (epidemic) propagation and their relative magnitudes.

Focusing specifically on gun violence is crucial because it is increasingly recognized as a public health crisis [26–28], being the most lethal form of violence and accounting for nearly 86% of homicides in Washington, D.C [29]., and approximately 76% nationally [30]. Additionally, gun violence incurs significant economic costs [31, 32] and sharp changes in overall violence trends are consistently driven by gun-related incidents [33, 34], making it essential to employ methods that effectively decompose endemic and episodic patterns in this context. Our study addresses existing gaps by applying this innovative modeling framework to weekly gun violence data at the census tract level in Washington, D.C. from 2014 to 2025. This approach enables the estimation of gun violence risk at fine spatial and temporal scales, effectively separating baseline neighborhood risk from within-area recurrence and between-area diffusion. As one of the first studies to use this type of spatiotemporal decomposition for firearm violence in a U.S. city, we provide novel insights into how structural SDoH and place-based vulnerabilities, alongside spatiotemporal interactions, drive both persistent and episodic patterns of gun violence.

## Methods

### Study Design and Data Sources

We analyzed weekly gun violence incident data geocoded to Washington, D.C. census tracts from January 2014 to July 2025, using an ecological study design to assess the association between tract-level sociodemographic and environmental factors and rates of gun violence. The study period spans just over a decade of weekly incident data, providing a sufficiently long time series for stable estimation of seasonal, endemic, and epidemic parameters. This window encompasses the COVID-19 pandemic, which we modeled as an explicit structural break rather than unmodeled noise. Gun violence incidents were identified from the ASAP (Analytical Services Application) crime report database provided by the District of Columbia Metropolitan Police Department (MPD), publicly available through Open Data DC [35], as any record where the method was listed as “Firearm,” encompassing homicides, assaults, and robberies regardless of fatality status. Incidents were further categorized by shift (midnight, evening, day) to examine temporal patterns. We maintained a unified outcome of all firearm-related incidents to capture the full spectrum of firearm-related harm, enhance statistical power for tract-week-level estimation, and reflect the interconnected nature of different incident types in urban settings.

We hypothesized that adverse socio-environmental and structural conditions, including socioeconomic vulnerability and high alcohol outlet density, would be the primary drivers of gun violence risk, and that short-term epidemic dynamics would contribute a smaller but spatially concentrated share of incident variability.

### Explanatory Variables

Tract-level predictors included sociodemographic and environmental characteristics updated across multiple time points (time-varying) or measured at a single point in time (fixed). The CDC’s Social Vulnerability Index [36] provides a composite measure of community vulnerability to external stressors derived from the American Community Survey; we used five releases (2014, 2016, 2018, 2020, 2022) of the overall SVI and four thematic indices: (1) Socioeconomic Status (SES), capturing poverty, unemployment, housing cost burden, and educational attainment; (2) Household Characteristics (HHC), reflecting disability, single-parent households, language isolation, and age structure; (3) Racial and Ethnic Minority Status, reflecting minority population concentration and limited English proficiency; and (4) Housing Type and Transportation (HTT), capturing crowded housing, mobile homes, multi-unit structures, and vehicle access. Each theme is scored from 0 to 1, with higher values indicating greater vulnerability; tract-level scores were linked to the nearest available release year. Prior to modeling, each SVI theme was divided by 10 so that model coefficients represent the change in risk associated with a 0.1-unit increase on the index. The Reinvestment Fund’s Limited Supermarket Access (LSA) designations (2013, 2017, 2022) [37], tree canopy density (2020) [38], bus stop density (2023) [39], alcohol outlet (any business with a license to sell or serve beer, wine, and/or liquor) density from DC’s Alcoholic Beverage and Cannabis Administration (2024) [40], and street light density from DC’s Department of Transportation (2024) [41] were each measured at a single point in time and treated as fixed tract-level characteristics throughout the study period. We also included a categorical variable for COVID-19 phases: “Pre” (January 2014–March 10, 2020), “Quarantine/Lockdown” (March 11, 2020–June 11, 2021), and “Post” (June 12, 2021 onward). A geographic crosswalk [42] reconciled differences between 2020 census tracts (*n* = 206) and 2010 tract boundaries (*n* = 179), resulting in 106,684 tract-week observations (179 tracts × 596 weeks). Supplemental materials include maps showing the spatial distribution of each explanatory variable.

### Statistical Analysis

We first conducted exploratory analyses using Zero-Inflated Negative Binomial (ZINB) regression to account for overdispersion and excess zeros typical in weekly gun violence counts. This approach helped distinguish tracts with frequent incidents from those with fewer occurrences and informed the selection of covariates for subsequent modeling. Model performance was compared using Akaike Information Criterion (AIC), confirming ZINB superiority over Poisson variants. Pearson residuals were examined as a diagnostic for extreme lack of fit; a conservative threshold of |r|>20 was used to flag observations with exceptionally large discrepancies between observed and expected counts rather than as a formal goodness-of-fit criterion. Fewer than 0.02% of tract-week observations exceeded this threshold, indicating that extreme departures from model expectations were rare. A random intercept for census tract was included to capture unobserved local heterogeneity, and the negative binomial distribution accommodated remaining variability. The ZINB model served as an exploratory screening step to identify predictors with meaningful associations with gun violence counts prior to main model fitting. Variables with statistically significant associations (*p* < 0.05) and meaningful representation of the structural conditions hypothesized to drive chronic risk were candidates for inclusion in the endemic component.

Following this preliminary exploration, our main analysis utilized an endemic-epidemic modeling approach [43] to capture both persistent long-term (endemic) and short-term (epidemic) dynamics of gun violence events from 2014 to 2025. This framework was selected because it decomposes observed counts into endemic and epidemic components within a single regression model, enabling simultaneous estimation of covariate effects on long-term risk and formal quantification of within-tract recurrence and between-tract spillover. This directly addresses our central aim of distinguishing the structural conditions that sustain chronic risk from the short-term processes that drive episodic surges. Weekly counts ${Y}_{i,t}$ for tract *i* at time *t* were modeled using a negative binomial distribution conditional on the past, where the conditional mean was decomposed as:

$${\mu}_{i,t}={e}_{i,t}{v}_{i,t}+{\lambda}_{i,t}{Y}_{i,t-1}+{\phi}_{i,t}\sum_{j\ne i}{w}_{ij}{Y}_{j,t-1}$$

The endemic component ${v}_{i,t}$ represents baseline risk and incorporates tract-level temporal trends, seasonal effects, and tract-level covariates offset by tract-level population ${e}_{i,t}$, reflecting long-term patterns influenced by chronic socio-environmental conditions. The epidemic component captures short-term dependence on prior incidence and includes two distinct processes: (1) within-tract autoregressive effects (${\lambda}_{i,t}$), modeled using lagged counts from the same tract ${Y}_{i,t-1}$, and (2) between-tract spatial spillover effects (${\phi}_{i,t}$) modeled using spatially weighted lagged counts from adjacent tracts (binary first-order adjacency matrix based on queen contiguity, i.e., shared boundary or vertex) $\sum_{j\ne i}{w}_{ij}{Y}_{j,t-1}$. This decomposition allows separation of background risk from localized temporal persistence and spatial diffusion. Raw incident counts rather than rates were used as the outcome: the population offset in the endemic component adjusts baseline risk for population heterogeneity, so endemic covariate effects are interpretable on the rate scale, while the epidemic component operates on raw lagged counts, consistent with the hypothesis that short-term contagion scales with the number of recent events rather than rates. Overdispersion was handled through the negative binomial likelihood, while the high frequency of zero counts was accommodated naturally by low expected means in low-risk tract-weeks and the conditional epidemic structure, rather than through explicit zero inflation.

Continuous built-environment variables (streetlight, bus stop, and alcohol outlet densities) were log-transformed to reduce skewness. We utilized the hhh4 [43] package for the endemic-epidemic model in R v4.4.2 [44], and all maps were produced in ArcGIS Pro v3.6.0 [45]. Supplemental materials include maps illustrating the spatial distribution of gun violence incidents and explanatory variables.

### Ethical Considerations

All data used in this analysis were deidentified and publicly available; as such, the study was exempt from institutional review board review.

### Data Availability

Data will be made available on Fig Share pending publication. Link is masked for peer-review.

## Results

### Descriptive Statistics

Table 1 provides a comprehensive overview of the characteristics of gun violence outcomes in Washington, D.C. and associated explanatory variables across 106,684 tract-week observations from January 2014 to July 2025. A total of 23,971 firearm-related incidents were recorded, with an average of 73.4 incidents per week. By incident type, the weekly average included approximately 14.3 firearm-related assaults, 2.7 firearm-related homicides, 21.8 firearm-related robberies, and 1.1 other firearm-related events. The average population within each census tract was 3,576.41, with a social vulnerability index (SVI) value averaging 0.49, reflecting varied levels of minority status, socioeconomic factors, household composition, and housing types.

**Table 1:** Summary characteristics of gun violence incidents in Washington, D.C. (January 2014-July 2025) and explanatory variables

Outcome	Mean (Min–Max)
Incidents (counts) per week	73.4 (13–249)
Firearm-Related Assaults per Week	14.3 (4–35)
Firearm-Related Homicides per Week	2.70 (0–15)
Firearm-Related Robberies per Week	21.8 (3–93)
Other Firearm-Related Events per Week	1.11 (0–9)
Population and social vulnerability	Mean (Min–Max)
Population Total	3576.41 (50.07–6542.42)
Overall SVI	0.49 (0–0.99)
SVI Minority Status and Language	0.51 (0–0.92)
SVI Socioeconomic Status	0.51 (0–0.99)
SVI Household Composition	0.51 (0–0.99)
SVI Housing Type and Transportation	0.49 (0–0.99)
Built environment	Mean (Min–Max)
Tree canopy cover (% of tract)	29.81% (6.20%−80.97%)
Streetlight density (Sq. Km per tract)	587.20 (13.97–1131.55)
Bus stop density (Sq. Km per tract)	25.15 (0.41–75.27)
Supermarket access	Proportion of tracts (*n* = 179)
Limited Supermarket Access Area (% of tracts)	18.44%
Low Access/Low Population (% of tracts)	31.28%
Not an LSA Area (% of tracts)	50.28%
Alcohol environment	Outlets per 10,000 people
Alcohol outlet density	54.90 (0–2396.78)
Temporal factors	
Season	Total incidents (%)
Fall	6292 (26.25%)
Spring	5386 (22.47%)
Summer	6761 (28.20%)
Winter	6292 (26.25%)
COVID-19 Period	Total incidents (%)
Pre-Lockdown (January 2014 to March 10, 2020)	11,391 (47.52%)
Lockdown (March 11, 2020, to June 11, 2021)	2533 (10.57%)
Post-Lockdown (June 12th, 2021-onwards)	10,047 (41.91%)
Police shift	Total incidents (%)
Day Shift	4013 (16.74%)
Evening Shift	8362 (34.88%)
Overnight Shift	11,596 (48.38%)

Average tract-level tree canopy area proportion was 29.81%, while streetlight density is approximately 587.20 per square kilometer, and bus stop density averages 25.15 per square kilometer. Regarding supermarket access, 18.44% of tracts fall within limited supermarket access areas, while 31.28% are classified as low access/low population areas, and approximately 50% of tracts were not designated as a limited supermarket access area. The average alcohol outlet density is notably high at 54.90 outlets per 10,000 people. Temporal factors affecting gun violence are also highlighted; incidents peak during the summer months, accounting for 28.20%, while the COVID-19 pre-lockdown period represents 47.52% of total incidents recorded from January 2014 to March 2020. The COVID-19 lockdown period contained 10.57% of incidents, and the post-lockdown period represented 41.91% of all gun violence incidents. Furthermore, the distribution of incidents varies by police shift, with overnight shifts experiencing the highest frequency at 48.38%, compared to 34.88% during evening shifts and 16.74% during day shifts.

We also summarized gun violence outcomes by Social Vulnerability Index (SVI) quartile from 2014 to 2025 (Supplemental Table 1). As the SVI increases from very low to very high quartiles, there is a corresponding rise in the average number of incidents per week, from 4.54 in the very low quartile to 17.5 in the very high quartile. Regarding firearm-related assaults, the very low SVI quartile included 0.87 per week, while the very high quartile saw 7.74 per week. Similarly, for firearm-related homicides, the very low quartile saw 0.14 per week compared to 1.54 in the very high quartile. Firearm-related robberies yielded 3.28 per week across D.C. in the very low SVI quartile and 8.15 in the very high quartile. Lastly, for other firearm-related events, the very low SVI quartile comprised 0.05 per week, and 0.14 in the very high quartile.

### Exploratory ZINB Model Results

Table 2 displays the results from the Zero-Inflated Negative Binomial (ZINB) model, highlighting significant associations between gun violence incidents and a range of influencing socio-environmental factors across different tracts in DC. The results indicate that tracts in the “Very High” category of the SVI socioeconomic status (SES) theme, reflecting the greatest socioeconomic disadvantage, had significantly higher rates of gun violence compared to tracts in the “Very Low” (least vulnerable) reference category (RR = 1.188, 95% CI 1.069–1.319, *p* < 0.001). Similarly, tracts designated as Limited Supermarket Access Areas were associated with increased gun violence (RR = 1.581, 95% CI 1.349–1.852, *p* < 0.001), as were tracts classified as Low Access/Low Population areas (RR = 1.188, 95% CI 1.043–1.353, *p* = 0.010), compared to tracts not designated as limited access areas. Inversely, tree canopy density is associated with a reduction in gun violence (RR = 0.978, 95% CI 0.971–0.986, *p* < 0.001), while bus stop density has a positive association (RR = 1.012, 95% CI 1.003–1.021, *p* = 0.007). Alcohol outlet density shows a strong positive relationship with gun violence (RR = 2.312, 95% CI 1.181–4.528, *p* = 0.014), whereas higher streetlight density is associated with decreased rates of gun violence (RR = 0.388, 95% CI 0.216–0.699, *p* < 0.001). Seasonal effects reveal increased gun violence in, summer (RR = 1.095, 95% CI 1.061–1.130, *p* < 0.001), fall (RR = 1.092, 95% CI 1.058–1.127, *p* < 0.001), and decreased gun violence in spring (RR = 0.943, 95% CI 0.913–0.975, *p* < 0.001) compared to winter. Midnight and evening police shifts were strongly associated with gun violence (RR = 15.207, 95% CI 14.777–15.649 and RR = 14.482, 95% CI 14.039–14.939, respectively; *p* < 0.001 for both), though shift reflects the temporal distribution of incident reporting rather than a tract-level structural condition and was therefore excluded from the endemic-epidemic model. Additionally, during the COVID-19 lockdown period, gun violence significantly decreased (RR = 0.363, 95% CI 0.347–0.379, *p* < 0.001), with a similar reduction observed in the post-lockdown period (RR = 0.426, 95% CI 0.415–0.438, *p* < 0.001).

**Table 2:** Adjusted rate ratios (RRs) and 95% confidence intervals from the zero-inflated negative binomial model of gun violence events, Washington, D.C. (2014–2025)

Variable	RR	95% CI	***p***-value
SVI: Socioeconomic Status (SES) Theme (Ref = Very Low)			
SVI SES Low	0.979	0.909–1.054	0.572
SVI SES High	1.08	0.982–1.188	0.113
SVI SES Very High	1.188	1.069–1.319	< 0.001
**SVI: Housing Type and Transportation (HTT) Theme (Ref = Very Low)**			
SVI HTT Low	1.069	1.006–1.135	0.031
SVI HTT High	0.993	0.931–1.059	0.831
SVI HTT Very High	0.941	0.876–1.011	0.096
**SVI: Household Characteristics (HHC) Theme (Ref = Very Low)**			
SVI HHC Low	0.95	0.888–1.016	0.132
SVI HHC High	0.965	0.886–1.050	0.405
SVI HHC Very High	1.059	0.966–1.160	0.22
**Supermarket Access (Ref = Not a Limited Access Area)**			
Low Access/Low Population	1.188	1.043–1.353	0.01
Limited Supermarket Access Area	1.581	1.349–1.852	< 0.001
**Tree Canopy Density**	0.978	0.971–0.986	< 0.001
**Bus Stop Density**	1.012	1.003–1.021	0.007
**Alcohol Outlet Density**	2.312	1.181–4.528	0.014
**Street Light Density**	0.388	0.216–0.699	0.002
**Season (Ref = Winter)**			
Spring	0.943	0.913–0.975	< 0.001
Summer	1.095	1.061–1.130	< 0.001
Fall	1.092	1.058–1.127	< 0.001
**Police Shift Proportion of Cases (Ref = Day Shift)**			
Midnight	15.207	14.777–15.649	< 0.001
Evening	14.482	14.039–14.939	< 0.001
**COVID-19 Period (Ref = Pre-Lockdown Period)**			
Lockdown/Quarantine	0.363	0.347–0.379	< 0.001
Post-Lockdown/Quarantine Period	0.426	0.415–0.438	< 0.001

SVI themes were categorized into quartiles based on the national distribution of tract-level scores: Very Low (0–0.25), Low (> 0.25–0.50), High (> 0.50–0.75), Very High (> 0.75–1.00). Higher SVI scores indicate greater social vulnerability. The SVI Socioeconomic Status (SES) theme captures poverty, unemployment, housing cost burden, and educational attainment; higher scores reflect greater socioeconomic disadvantage. All RRs for categorical variables reflect comparisons to the respective reference category (Very Low); RRs for continuous variables (tree canopy density, bus stop density, alcohol outlet density, street light density) reflect a one-unit increase in the log-transformed variable

### Endemic-Epidemic Modeling Results (Main Analysis)

Table 3 presents the parameter estimates from the endemic–epidemic model of gun violence events in Washington, D.C. from 2014 to 2025. In terms of endemic components, the autoregressive (AR) parameter is markedly high at 6.99 (95% CI 5.92–8.25, *p* < 0.001), indicating a strong persistence of baseline risk of gun violence from prior weeks. Furthermore, approximately 79% of gun violence was attributed to the endemic component. Limited supermarket access is associated with increased gun violence (RR = 1.38, 95% CI 1.34–1.42, *p* < 0.001), while tree canopy density is inversely related (RR = 0.98, 95% CI 0.98–0.99, *p* < 0.001). Streetlight density shows a non-significant trend (RR = 0.83, 95% CI 0.67–1.04), whereas bus stop density was positively associated with gun violence (RR = 1.36, 95% CI 1.30–1.42, *p* < 0.001). Alarmingly, alcohol outlet density presents a very high rate ratio (RR = 11.55, 95% CI 9.46–14.10, *p* < 0.001) and both the SVI SES theme (RR = 1.16, 95% CI 1.14–1.17, *p* < 0.001) and household characteristics theme (RR = 1.09, 95% CI 1.03–1.15, *p* < 0.001) were strongly associated with increased gun violence, where RRs reflect the change in risk associated with a 0.1-unit increase on the index (e.g., from SVI = 0.6 to 0.7). The COVID-19 quarantine (RR = 0.34, 95% CI 0.31–0.37, *p* < 0.001) and post-quarantine periods (RR = 0.38, 95% CI 0.35–0.42, *p* < 0.001) were associated with significant decreases in gun violence risk.

**Table 3:** Rate ratios (RRs) and 95% confidence intervals for covariate effects and endemic and epidemic component parameters from the endemic–epidemic model of weekly gun violence incidents across 179 census tracts in Washington, D.C. (2014–2025)

Variable	RR	95% CI
Endemic components		
Parameter ${v}_{i,t}$	6.99	5.92–8.25*
Supermarket access	1.38	1.34–1.42*
Tree canopy density	0.98	0.98–0.99*
Street light density	0.83	0.67–1.04
Bus stop density	1.36	1.30–1.42*
Alcohol outlet density	11.55	9.46–14.10*
SVI: socioeconomic status	1.16	1.14–1.17*
SVI: household characteristics	1.09	1.03–1.15*
COVID-19 quarantine period	0.34	0.31–0.37*
COVID-19 post quarantine period	0.38	0.35–0.42*
Epidemic components		
Within-tract AR parameter ${\lambda}_{i,t}$	0.02	0.01–0.02*
Spatial AR parameter ${\phi}_{i,t}$	0.023	0.021–0.025*
Epidemic AR parameter (same tract, temporal lag)	0.11	0.11–0.12*
COVID-19 quarantine period	1.09	0.75–1.59
COVID-19 post quarantine period	1.07	0.83–1.36
Seasonal effect parameters		
Sine	1.01	0.98–1.04
Cosine	0.98	0.95–1.00

For continuous built-environment variables (tree canopy density, bus stop density, alcohol outlet density, street light density), RRs reflect a one-unit increase in the log-transformed variable. RRs for SVI themes reflect the change in risk associated with a 0.1-unit increase on the index (e.g., from SVI = 0.6 to 0.7). For categorical variables, RRs reflect each category compared to the stated reference group. Endemic component RRs reflect associations with baseline (long-term) gun violence risk; epidemic component parameters capture short-term within-tract and between-tract dynamics

In the epidemic component, the within-tract AR parameter ${{\boldsymbol{\lambda}}}_{{\boldsymbol{i}},{\boldsymbol{t}}}$ was low (RR = 0.02, 95% CI 0.01–0.02, *p* < 0.001), indicating minimal influence of prior counts within the same tract on current gun violence events. The spatial AR parameter ${\phi}_{i,t}$ was also low (RR = 0.023, 95% CI 0.021–0.025, *p* < 0.001), indicating minimal influence of neighboring tracts on current gun violence events. The temporal AR parameter in the epidemic component indicates minimal persistence of gun violence from one week to the next within the same tract (RR = 0.11, 95% CI 0.11–0.12). The COVID-19 quarantine (RR = 1.09, 95% CI 0.75–1.59) and post-quarantine periods (RR = 1.07, 95% CI 0.83–1.36) show no significant increase in gun violence relative to the endemic period. Seasonal parameters yielded non-significant results for both the sine (RR = 1.01, 95% CI 0.98–1.04) and cosine components (RR = 0.98, 95% CI 0.95–1.00).

**Fig. 1:**
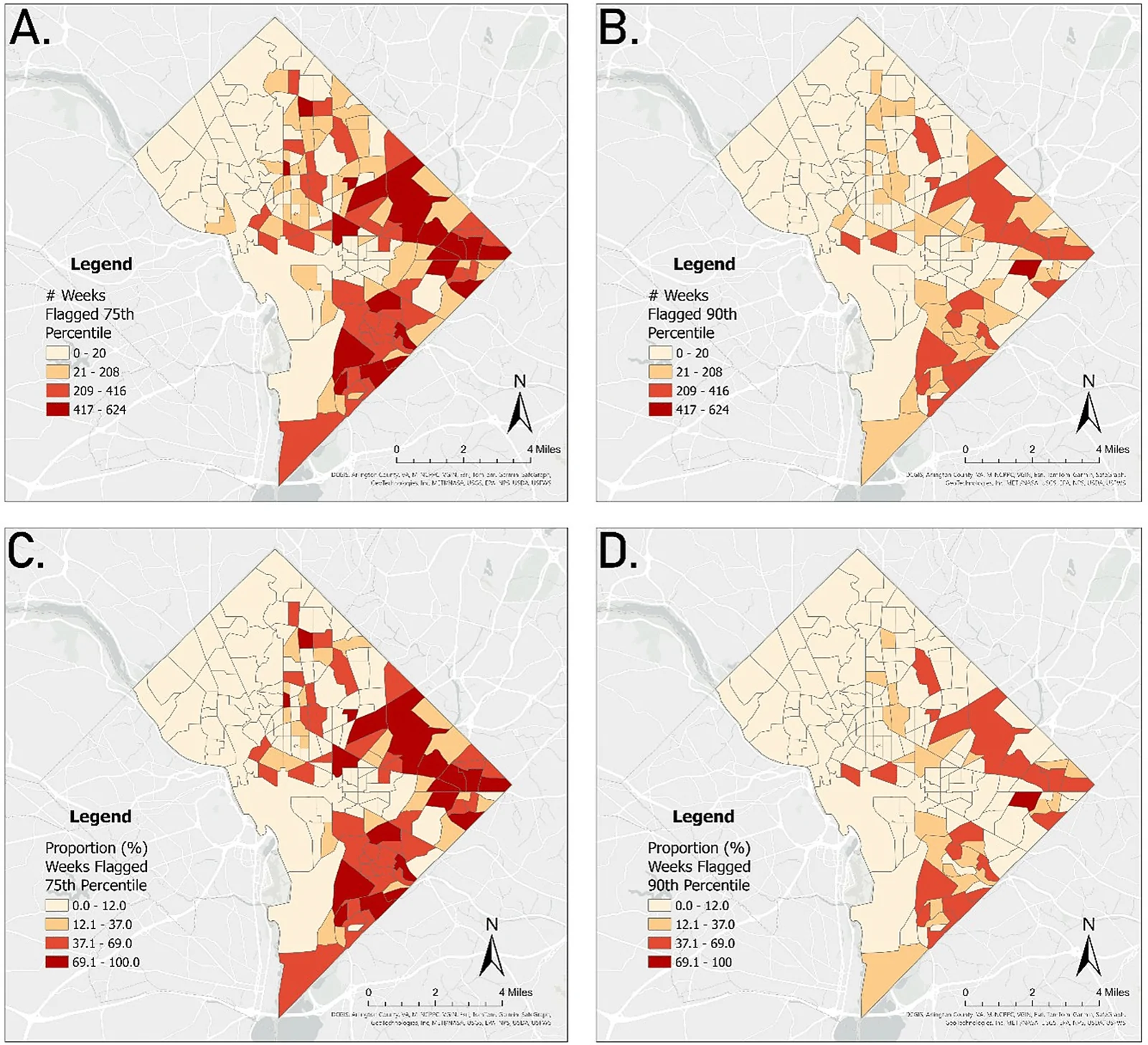
The number (**A**, **B**) and proportion (**C, D**) of weeks in which gun violence incidents exceeded the 75th and 90th percentile thresholds across Washington, D.C

To further operationalize “epidemic” gun violence activity at the tract-week level, we defined epidemic thresholds using the 75th and 90th percentiles of all fitted values from our endemic-epidemic model (Fig. 1). These thresholds capture extreme temporal surges in model-predicted gun violence, irrespective of spatial differences in baseline risk. For each census tract, we calculated the number (Fig. 1A, B) and proportion (Fig. 1C, D) of weeks where the fitted count exceeded these thresholds, identifying areas experiencing sustained or recurrent surges above expected levels rather than simply high average violence. Tracts in the southeastern quadrant consistently accumulated the greatest number of flagged weeks at both thresholds, and several exceeded the 75th percentile threshold in more than 69% of all study weeks, suggesting that elevated violence is the persistent norm in these areas rather than an episodic deviation. Together, the four panels separate tracts experiencing frequent and intense epidemic activity from those with only occasional surges, providing a spatially explicit basis for prioritizing place-based intervention and adapting the threshold framework for prospective surveillance.

## Discussion

Our analysis reveals that gun violence in Washington, D.C. primarily reflects steady, place-based risk, with approximately 79% of incidents attributed to endemic factors driven by persistent neighborhood conditions. These structural factors, such as socioeconomic vulnerability and access to resources, are essential for understanding long-term patterns of violence. In contrast, the remaining ~ 21% of gun violence incidents are characterized as short-term spikes, which occur near recent incidents and are brief and highly localized, exhibiting little spillover into other neighborhoods. This indicates that while high baseline (endemic) risk identifies general areas of concern, it does not effectively predict where these spikes will occur, as they are more directly influenced by recent nearby incidents rather than overall neighborhood risk levels.

The analysis also identified that higher bus stop density was associated with increased endemic risk of gun violence in D.C. Conversely, neighborhoods with more tree cover demonstrated slightly lower gun violence rates, supporting findings in environmental criminology that suggest green spaces and urban beautification can contribute to reduced violence or foster safer environments. Increased tree cover may enhance social cohesion, foster community pride, and improve perceived safety among residents [46–48]. This aligns with recent work which found that tree canopy coverage was associated with lower crime rates in D.C., although this association was weakest in communities marked by high poverty and low homeownership [48]. Tree canopy coverage is also not independent of historical structural processes. Neighborhoods with greater tree cover tend to have experienced less severe redlining, fewer racially exclusionary housing policies, and greater cumulative investment, meaning canopy may partly reflect the legacy of structural advantage rather than a direct causal mechanism [49]. The observed inverse association likely operates through multiple concurrent pathways, and caution is warranted in interpreting it as evidence that greening alone would reduce violence without addressing underlying structural conditions.

The associations between SVI themes and relative risk align with the understanding that socioeconomic disparities play a critical role in shaping gun violence outcomes [50]. This suggests that while short-term interventions, such as hot spot policing [51], are often implemented to combat surges in violence, they may not adequately address the underlying and persistent factors that contribute to gun violence over time given our finding that approximately 80% of community gun violence was endemic. Furthermore, the weak spatial spillover effect found in our study highlights the importance of localized interventions beyond policing, as the influence of gun violence in one neighborhood on adjacent areas may be less significant than expected.

Periods of unprecedented public health and social disruption such as the pandemic, can reshape the dynamics of violence by altering patterns of community presence, policing, and social cohesion [52]. The COVID-19 periods represented a significant disruption in social dynamics, impacting human interactions and mobility, which are relevant for epidemic spikes in violence. However, our findings suggest that the impact of these changes was not significant enough to alter the underlying mechanisms driving epidemic violence in D.C. More broadly, the temporal structure of our model distinguished stable seasonal and long-term endemic patterns from short-term epidemic fluctuations. Seasonal patterning was evident in our exploratory model, with violence elevated in spring, summer, and fall relative to winter, consistent with prior evidence on seasonal patterning of violence. In the endemic-epidemic model, the epidemic component showed minimal week-to-week persistence and non-significant seasonal parameters, indicating that short-term contagion dynamics were neither strongly self-sustaining nor seasonally driven. This separation underscores that the temporal signal in gun violence is carried largely by stable, structurally patterned baseline risk rather than by episodic surges.

Among the significant explanatory factors identified, alcohol outlet density exhibited the strongest association with gun violence incidents. While our design is associational and does not support causal inference, this finding is consistent with and adds evidence to a body of research linking alcohol availability to violence, strengthening the case for examining outlet density and operational conditions as policy levers. Prior work suggests that limiting the number of alcohol outlets in vulnerable neighborhoods and enforcing stricter licensing regulations may be associated with reduced violence [53]. Future work should examine how the hours of operation, outlet type (e.g., off- vs. on-premise), and license type potentially influence gun violence risk and spillover into nearby city blocks or neighborhoods.

Gun violence is often concentrated into areas where, through policy action or inaction, disadvantage and disenfranchisement have also been concentrated [54]. Addressing these upstream or ‘root cause’ factors are an essential element of a public health approach to gun violence. However, interventions aimed at enhancing neighborhood socioeconomic conditions typically require substantial time and investment to lead to substantive impacts on violence rather than perpetuating harms [55]. To meaningfully address violence, efforts must consider both structural and social determinants of health while also focusing on dynamic and proximate processes (such as local recurrence, spillover, and situational opportunity structures). Still, the predominance of endemic factors should not be read as a fixed or unavoidable link between poverty and violence. The relationship between socioeconomic disadvantage and violence is shaped by modifiable structural conditions, including disinvestment, segregation, and unequal access to resources, rather than by disadvantage itself, and varies across contexts rather than following a single fixed pattern [56, 57].

### Limitations

This study is not without limitations. First, the data used in our analysis primarily reflects incidents known to police, which may lead to underreporting or discrepancies in true gun violence rates, particularly in areas with less community engagement or poorer relationships with law enforcement. Second, the reliance on secondary data sources means we could not control for all possible confounding variables that may influence the relationships observed in our study. Third, several built-environment variables (tree canopy density, bus stop density, street light density, and alcohol outlet density) were measured at a single point in time and treated as fixed throughout the study period; as such, their coefficients reflect time-averaged associations across the study period rather than true within-tract change. This may modestly affect the endemic component estimates for these predictors, though it is unlikely to alter the overall finding that endemic factors dominate the variance decomposition, as that finding is primarily driven by the SVI and supermarket access variables, which were updated across multiple time points. Fourth, the CDC SVI is derived from American Community Survey multi-year estimates, meaning that successive SVI releases share overlapping survey years. This reduces year-to-year independence across SVI time points and may introduce modest smoothing in the time-varying covariate series. Finally, while our modeling approach captures spatiotemporal dynamics at the census tract level, it may not fully reflect finer-scale contextual nuances or the impact of evolving social policies on gun violence over time.

## Public Health Implications

Our findings highlight the need for strategies that move beyond reactive enforcement and address the structural and social determinants underlying firearm violence. Traditional policing may temporarily suppress incidents but does not change the neighborhood conditions that sustain them. Long-term strategies require harm reduction approaches that expand access to education, housing, employment, and community-based services, along with investments in green space and neighborhood infrastructure to strengthen community resilience and promote health equity.

The use of high-resolution spatiotemporal modeling provides public health agencies with an actionable framework for identifying when and where violence is most likely to occur. By capturing localized spikes and persistent structural risks at the census tract level on a weekly basis, this approach supports timely, place-based prevention efforts. These findings underscore the importance of shifting resources toward proactive, community-centered interventions that prioritize public health, stability, and safety over punitive enforcement. This comprehensive approach is crucial for addressing the root causes of gun violence and fostering safer communities.

## Supplementary Information

Below is the link to the electronic supplementary material.

ESM 1 (DOCX 3.12 MB)

ESM 2 (DOCX 14.9 KB)
