# Supplementary material for: Enhancing Gun Violence Surveillance with Endemic-Epidemic Modeling: A Case Study in Washington, D.C
Source: J Urban Health. 2026 Aug 17;103(5):1045–57. doi: 10.1007/s11524-026-01128-5 (PMC13533284; doi:10.1007/s11524-026-01128-5)
Supplement: Supplementary file 2 [file 11524_2026_1128_MOESM2_ESM.docx]

**Supplemental Table 1. Gun violence incident types by SVI quartile in Washington, D.C., Jan 2014–Jul 2025.** **Counts (n) and percentages reflect incidents per quartile; weekly averages presented for each incident type**

|  | **SVI Quartile** | | | | |
| --- | --- | --- | --- | --- | --- |
| **Outcome** | **Very Low (n=2,586, 11.84%)** | **Low (n=4,143, 17.17%)** | | **High (n=6,651, 27.57%)** | **Very High (n=10,474, 43.42%)** |
| Incidents per week | 4.54 | 7.01 | 11.1 | | 17.5 |
| Total Firearm-Related Assaults (n=8,524) | 0.87 | 1.87 | 3.82 | | 7.74 |
| Total Firearm-Related Homicides (n=1,619) | 0.14 | 0.32 | 0.71 | | 1.54 |
| Total Firearm-Related Robberies (n=13,466) | 3.28 | 4.63 | 6.53 | | 8.15 |
| Total Other Firearm-Related Events (n=215) | 0.05 | 0.08 | 0.09 | | 0.14 |
