# Supplementary material for: Enhancing Gun Violence Surveillance with Endemic-Epidemic Modeling: A Case Study in Washington, D.C
Source: J Urban Health. 2026 Aug 17;103(5):1045–57. doi: 10.1007/s11524-026-01128-5 (PMC13533284; doi:10.1007/s11524-026-01128-5)
Supplement: Supplementary file 3 [file 11524_2026_1128_MOESM1_ESM.docx]

**Supplemental Figures**
